# The Transition to Minimal Consciousness through the Evolution of Associative Learning

**DOI:** 10.3389/fpsyg.2016.01954

**Published:** 2016-12-22

**Authors:** Zohar Z. Bronfman, Simona Ginsburg, Eva Jablonka

**Affiliations:** ^1^The Cohn Institute for the History and Philosophy of Science and Ideas, Tel Aviv UniversityTel Aviv, Israel; ^2^School of Psychology, Tel Aviv UniversityTel Aviv, Israel; ^3^Department of Natural Science, The Open University of IsraelRaanana, Israel; ^4^The Sagol School of Neuroscience, Tel Aviv UniversityTel Aviv, Israel

**Keywords:** evolution of associative learning, evolution of consciousness, the distribution problem, learning and consciousness, evolutionary transitions

## Abstract

The minimal state of consciousness is sentience. This includes any phenomenal sensory experience – exteroceptive, such as vision and olfaction; interoceptive, such as pain and hunger; or proprioceptive, such as the sense of bodily position and movement. We propose *unlimited associative learning* (UAL) as the marker of the *evolutionary transition to minimal consciousness (or sentience)*, its phylogenetically earliest sustainable manifestation and the driver of its evolution. We define and describe UAL at the behavioral and functional level and argue that the structural-anatomical implementations of this mode of learning in different taxa entail subjective feelings (sentience). We end with a discussion of the implications of our proposal for the distribution of consciousness in the animal kingdom, suggesting testable predictions, and revisiting the ongoing debate about the function of minimal consciousness in light of our approach.

“*Mind can be understood only by showing how mind is evolved*”

(Spencer, 1890, p. 291).

## Introduction

One way to study a major evolutionary change, such as the transition to consciousness, would be to discover a trait that is necessary for the transition. This would make it possible to identify the evolutionarily most elementary form of consciousness that is free of the baggage of later-evolved structures and processes. The transition from inanimate matter to life shares interesting conceptual parallels with the emergence of consciousness. We use the approach of the Hungarian theoretical chemist Gánti (1975) and Gánti et al. (2003) to the study of minimal life as a heuristic for the study of the evolutionary transition to consciousness (for a detailed discussion of this heuristics see Ginsburg and Jablonka, 2015).

Gánti started by compiling a list of properties that jointly characterize minimal life and constructed a toy model (the chemoton) instantiating them. He suggested that one of the capacities of a minimal life system could be used as a marker of the evolutionary transition to sustainable minimal life. His specific suggestion, which was later sharpened and developed by Szathmáry and Maynard Smith (1995), was that the capacity for *unlimited heredity* marks the transition from non-life to sustainable life: only a system capable of producing hereditary variants that far exceed the number of potential challenges it is likely to face would permit long-term persistence of traits and cumulative evolution. Moreover, a system enabling unlimited heredity requires that the information-carrying subsystem is maintained by self-sustaining metabolic dynamics enclosed by a membrane – features like those exhibited by a proto-cell, an acknowledged minimal living system. Hence, once a transition marker is identified it allows the “reverse engineering” of the system that enables it.

In a previous work, we followed Gánti’s example by presenting a list of properties deemed individually necessary and jointly sufficient for characterizing minimal consciousness. We identified a putative evolutionary transition marker to minimal consciousness and characterized it at the behavioral level (Bronfman et al., 2016). Here, we build on our previous work, and further develop our theory regarding the evolution of consciousness. We begin by briefly summarizing our approach and setting the theoretical background for our proposal. Next, we characterize the suggested transition marker at the functional level, showing that the system underlying it has all the properties that jointly characterize consciousness. We then compare our approach to other leading theories of consciousness. Next, we review the distribution of the suggested transition marker in the animal kingdom, and conclude that it has emerged independently in three animal phyla. Finally, we point to the conceptual and ethical implications of our proposal.

## Characterizing Features of Minimal Consciousness

On the basis of a survey of studies of consciousness by philosophers of mind, psychologists, cognitive scientists and neuroscientists, we compiled a list of seven consciousness-characteristics that when jointly present, seem to capture the essence of minimal consciousness. Minimal consciousness refers to the most basic form of sensory phenomenal experience, such as seeing red (and experiencing redness), or feeling pain. This (basic) form of consciousness is distinct from high-order consciousness, which includes, in addition to the phenomenal experience itself, self-referential, or reflective (usually linguistic) content, such as the awareness of the thought that one is experiencing a red color (see discussion by Edelman, 2003; Block, 2005).

Although the emphases on the foundational properties of consciousness made by different investigators differ and the items on the list we present below are partially overlapping, this list represents a broad consensus (for a detailed discussion see Bronfman et al., 2016).

The characterizing features we identified are:

(1) Flexible value systems and goals that reflect or give rise to the motivational values of the organism’s ever-changing internal states and actions (e.g., Block, 1995; Panksepp, 2005; Denton, 2006; Damasio, 2010; Dickinson, 2010).
(2) Unity and diversity through sensory binding leading to the formation of a compound stimulus; the multiple underlying features of the compound are coherently and conjointly perceived, rather than each feature being perceived independently (see discussions in Tononi and Edelman, 1998; Engel et al., 2001; Llinás and Ribary, 2001; Crick and Koch, 2003).
(3) Global availability of information, involving multidirectional feedback and reentrant interactions that generate a state in which information is available to different specialized cognitive processes (Baars, 1993) that are otherwise “computationally isolated” (e.g., (Dehaene et al., 1998; Edelman and Tononi, 2000; Seth et al., 2005; Dehaene, 2014).
(4) Temporal thickness – the temporal persistence of mental states (e.g., James, 1890; Edelman, 1993; Lamme and Roelfsema, 2000; Crick and Koch, 2003; Shadlen and Kiani, 2013).
(5) Selection – involvement of processes of exploration and selective stabilization at different levels (neural, behavioral), including processes of action selection and selective attention (e.g., James, 1890; Changeux and Danchin, 1976; Edelman, 1987; Freeman, 2000; Merker, 2007; Fernando et al., 2010).
(6) Intentionality (aboutness) (e.g., Brentano, 1874; Searle, 1983; Freeman, 2000, 2003). There are processes of representation/referral; inputs from the body and the world are “mapped” onto dynamic perception and action models that are necessary for the constitution of phenomenal consciousness.
(7) Self and embodiment – no account of consciousness is possible without addressing the obvious fact that there is an agent that is sentient: it is the animal rather than its nervous system that is minimally conscious (for an insightful and detailed discussion see Merker, 2007, 2013). Various interactions of the brain with the physical body (beyond the brain), such as neuro-hormonal relations (e.g., Malenka et al., 2009), bioelectric fields (e.g., Levin, 2013) and neuro-immunological interactions (e.g., Schwartz and Kipnis, 2011) constitute the rich sense of self in animals. However, current models of agency or self-construction are still preliminary. We therefore focus here on the animal’s ability to form a representation of its body as distinct from the external world, yet embedded in it, as these dynamics are relatively well-understood, and are thought by many to lead to a sense of agency and “ownership” of the animal’s experiences (O’Regan and Noë, 2001; Merker, 2007, 2013; Thompson, 2007; Metzinger, 2009; Damasio, 2010; Seth, 2013; Seth and Friston, 2016).

The above “list” can be a basis for the construction a model of minimal consciousness which instantiates and generates the above features. We believe that at present we are far from such a generative model, although several models, based mainly on the mammalian system, have been suggested and are compatible with the list (e.g., Edelman, 2003; Freeman, 2003; Damasio, 2010; Dehaene, 2014), and so is the more general vertebrate-based model suggested by Merker (2007). A more roundabout way, which we adopt here, is to start from a characterization of an evolutionary transition marker of minimal consciousness as a first step toward “reverse engineering” the (minimally conscious) system that enables it.

## Unlimited Associative Learning, Its Functional Architectures and the Structures Implementing It

Our proposed evolutionary marker for the transition from non-sentient to sentient life is the capacity for unlimited associative learning (UAL) (Ginsburg and Jablonka, 2015; Bronfman et al., 2016). With UAL the number of associations among stimuli, and the number of possible reinforced actions that can be generated, is practically limitless. We begin by defining associative learning in general and then describe UAL at the behavioral, functional and structural levels. Next, we demonstrate how the various properties of the processes that generate the capacity for UAL parallel the properties of consciousness, and argue that sentience first emerged in the context of selection for UAL during the Cambrian era.

Associative learning (AL; see Macphail and Bolhuis, 2001 for a detailed discussion) involves the formation of a conditional (if-then) association between a conditioned stimulus (CS; e.g., the sound of a buzzer) and a reinforcing unconditioned stimulus (US) that elicits a physiological and motor response (e.g., food elicits salivation) (“Pavlovian” or “classical conditioning”), or between actions (e.g., pressing a lever) and their reinforcing outcomes (e.g., food) (“instrumental” or “operant conditioning”). However, in most conditions animals learn both about the world and their own behavior, a point which was stressed by early psychologists (reviewed in Rescorla and Solomon, 1967). Consider a case of classical conditioning when a particular cue (e.g., a particular smell) that predicts the presence of a prey elicits in the animal a reflex biting reaction (the UR). The actual reflex response must be tailored to the specific prey that the cue predicts (its size, its texture, etc.), and the animal learns both which cue predicts the prey and the modified UR (the CR) that the cue elicits. More generally, when the CR is not identical to the UR (it is a modification of it that is specific to the eliciting CS, which is very often the case when the UR is a locomotor-pattern), learning the CR is part of what the animal learns. With operant conditioning, the animal learns not only about its action (that pressing a lever is reinforcing) but also about the lever itself as a stimulus predicting reinforcement. We therefore prefer the conceptually clear (though idealized) distinction between self-learning (learning only about the consequences of one’s own actions – about *how* things are learned) and world learning (learning about *what* there is in the world through the reinforcing effects of relations between stimuli in the world that are independent of one’s own action; the distinction was suggested by Colomb and Brembs, 2010).

Whether learning about self or world or both, the stimuli that enter into conditional association can include “neutral” stimuli (which under ordinary conditions do not trigger a response), biologically important stimuli such as those involved in the maintenance of basic homeostatic and reproductive functions, the animal’s own actions, including stereotyped or novel actions, and the contexts in which particular stimuli and responses occur. Moreover, AL is, according to all definitions, “predictive.” Because the CS (e.g., the sound of a buzzer) repeatedly precedes the US (e.g., the smell of food), it comes to “predict” the response (salivation). The extent to which the organism anticipates the reinforcement when the CS occurs influences the strength of learning. Only unanticipated events lead to learning. The greater the difference between expected and actual reinforcement, the greater the differential learning success (this difference is called the prediction error (PE) (Rescorla and Wagner, 1972; Schultz et al., 1997; Schultz, 1998; Schultz and Dickinson, 2000). The blocking paradigm (Kamin, 1969), demonstrates how the presentation of a perfectly predicted reward does not generate a prediction-error and hence fails to generate new learning.

Here we focus on a specific form of world and self associative-learning that we call UAL, and distinguish it from simpler, more limited forms of associative learning. Although there are taxon-specific and ecology-specific constraints on the associability of different classes of compound stimuli, UAL greatly expands the range of stimuli and actions an animal can learn. We will argue that this expansion is generated by interacting mechanisms that construct a system whose properties are the same as those considered individually necessary and jointly sufficient for a system exhibiting minimal consciousness (which we listed above). We begin by defining UAL at the behavioral (overt) level, and then describe its underlying mechanisms and its functional architecture.

For UAL to occur, the following conditions must be met:

(i) The conditional stimulus (CS) or the reinforced actions are *compound* – i.e., consist of several features or action-patterns that are learned only as a whole, rather than separately. With compound associative learning (also known as spontaneous configuration; Razran, 1965; Bellingham and Gillette, 1981; or perceptual fusion; Konorski, 1967), an animal learns to associate a particular combination of color, shape, texture and smell to reinforcement (e.g., food). It thus learns to respond (e.g., salivate) upon future exposure to it, but it will not respond to each of the features when presented separately (e.g., only the shape) and will not even respond to a different spatial or temporal combination of the same features. An example of compound learning, also called non-elemental learning, occurs when the animal can learn that two different stimuli are associated separately with a negative reinforcement (A-, B-), whereas the compound stimulus is associated with a positive reinforcement (AB+) (Young et al., 2011). Another manifestation of non-elemental learning is discrimination learning, in which the animal learns to associate a particular reinforcement only with a specific compound pattern of stimuli (Watanabe et al., 1995; for sophisticated experiments showing the necessary role of the hippocampus in non-elemental learning in vertebrates, see Honey et al., 2014). Similarly, with compound instrumental learning a particular combination of action-patterns is learned by reinforcing the particular overall pattern, whereas a single action or a compound action made up of the same action patterns but in a different temporal order, is not.
(ii) The conditional stimulus or the reinforced actions are *novel* – i.e., they are neither reflex-eliciting nor pre-associated with a US or with past reinforcement. (For example, prior to learning, the sight of blue flowers with yellow spots does not elicit any observable aversive response.)
(iii) The learned conditional stimulus or the reinforced sequence of actions can subsequently support *second-order conditioning* (e.g., Rescorla and Holland, 1976; Gewirtz and Davis, 2000), acting as a US or as a reinforcement in future learning.

Note that according to the above description, many instances of AL *are not* UAL. For example, *Aplysia californica* is only capable of modifying the strength of preexisting associations between reflex-eliciting stimuli, and thus its mode of AL does not meet condition ii (see Carew et al., 1981; Hawkins et al., 1983; Brembs et al., 2002; Hawkins and Byrne, 2015 provide a review). The nematode *Caenorhabditis elegans* too does not fulfill the conditions for UAL: it has never been shown to be able to learn in a non-elemental manner; it can only form associations between each underlying feature of a compound stimulus and the reinforcement; hence it does not meet condition i. (For a discussion of further limitations of *C. elegans*’s capacity for learning see Bhatla, 2014.)

Three intertwined mechanisms are required for UAL: First, to enable compound learning, the compound (consisting of the specific configuration of the underlying elements or features) must be constructed via some form of intramodal and/or intermodal binding or feature-integration mechanisms that lead to the formation of a compound stimulus. In such a compound stimulus, multiple underlying features are coherently and conjointly experienced as opposed to each property/feature being perceived separately or in a different configuration. Binding requires multi-level hierarchical coding, with succeeding layers of the coding hierarchy increasing the specificity and quality of the percept (e.g., Lamme and Roelfsema, 2000; Senkowski et al., 2008). Furthermore, the hierarchical coding necessary for UAL must be *predictive*: as discussed above, studies of associative learning have shown that the strength of associative learning is not based on temporal contiguity alone. PE – the difference between actual and predicted reinforcement – is a key factor in modulating learning (Rescorla and Wagner, 1972; Schultz et al., 1997; Schultz, 1998; Schultz and Dickinson, 2000). One general theoretical account of this key concept is provided by hierarchical predictive coding theory, according to which one of the organism’s biggest challenges is to infer the (hidden) world-causes that give rise to the (observable) sensory signals the animal receives (Summerfield et al., 2006; Friston, 2009; Clark, 2013, 2015; Hohwy, 2013). To achieve this, the most efficient strategy is to constantly compare signals arriving from ‘low-level’ sensors (bottom–up information) with expectations generated at higher levels of processing (top–down, prior probabilistic models). Any discrepancy between the expected and actual signals is a prediction-error that leads to an adjustment of the expectations at the higher level. Thus, the organism is continuously predicting sensory signals and minimizing the discrepancy between its predictions and the actual signals. Such computations are implemented by assuming bi-directional connections (feed-forward and feedback) between two or more neural ensembles or units at different hierarchical levels.

Another equally fundamental type of PE is related to prospective actions. Any mobile animal must be able to distinguish between the sensory effects of its own actions and those originating from the outside world, independent of its actions: it must be able to predict the sensory effects of its actions and discount them. A relatively simple strategy is seen in animals such as nematodes or sea slugs (which can only reinforce stereotypic innate actions): a copy of the motor command that is sent to the muscles is also sent as an inhibitory signal, called “efferent copy” or “corollary discharge,” to sensory neural units, so that the animal inhibits the reflex reaction that its own movements would otherwise elicit (Holst and Mittelstaedt, 1971; Brembs and Heisenberg, 2000; Brembs, 2008; Craspe and Sommer, 2008). Hence, as with blocking, this reafference process can be implemented at the level of peripheral circuits. However, even an animal that can learn about association of only reflex-bound, non-compound stimuli must be able to block *conditioned* responses. In animals that exhibit complex and variant trains of actions, reafference must be based on *the updating of proprioceptive models.* (Peripheral and central reafference are reviewed in Craspe and Sommer, 2008; for a general discussion of reafference in the context of the predictive hierarchical coding framework see Hohwy, 2013; Clark, 2015; see Merfeld, 2001 for an early discussion of reafference and conscious body dynamics).

Second, with UAL reinforcement cannot be based only on the inherent reward/punishment value of the inputs or actions because, by definition, the valence or “value” of a novel compound stimulus or action is underdetermined by innate factors alone. The reinforcement/value system must take into consideration the overall homeostatic state of the animal. We use the term “value” in Krichmar’s and Edelman’s sense, to denote “neural structures that are needed for an organism to modify its behavior according to the salience of an environmental cue” (Krichmar and Edelman, 2002, p. 818; the “value” of a stimulus or an action should therefore be understood in a third-person sense). Moreover, the value-system must accommodate the ability for second-order learning, with each learned association sub-serving future learning of additional novel stimuli and actions. When occurring in the context of the ability for compound learning of novel stimuli and actions, second-order conditioning allows highly flexible adaptations to new environments (see discussion and examples in Bronfman et al., 2016). In mammals, the value system is implemented by several midbrain and cortical circuits and is based on dopamine neurons. These neurons signal reward prediction-error in a temporally precise manner, For example, in the ventral tegmental area (VTA) dopamine neurons are (i) excited by unexpected reward; (ii) unaltered by expected reward; and (iii) inhibited when expected reward is omitted (Schultz et al., 1997). In addition, the strength of their response correlates positively with the magnitude of the PE (see Schultz, 2013, for a review; for a discussion of the role of dopamine in aversive fear learning see Abraham et al., 2014).

Third, a distinct memory system for storing and recognizing compound patterns must be in place. Such dedicated memory systems have been identified in vertebrates, arthropods and cephalopods and some homologous structures have been observed in annelids, platyhelminths and nemerteans and even aceols (Strausfeld and Hirth, 2013; Wolff and Strausfeld, 2016). Such systems are necessary for memory-based predictive coding that requires the retrieval of memory traces (engrams) of associatively learned compound patterns upon which the perceptual expectation is reconstructed. A putative memory-based predictive-coding retrieval mechanism was recently identified in the human hippocampus, where engrams of compound patterns are identified (or completed, in situations in which the input is partial), and then sent to the cortex in order to construct perceptual predictions regarding the sensory inputs (Hindy et al., 2016).

**FIGURE 1:**
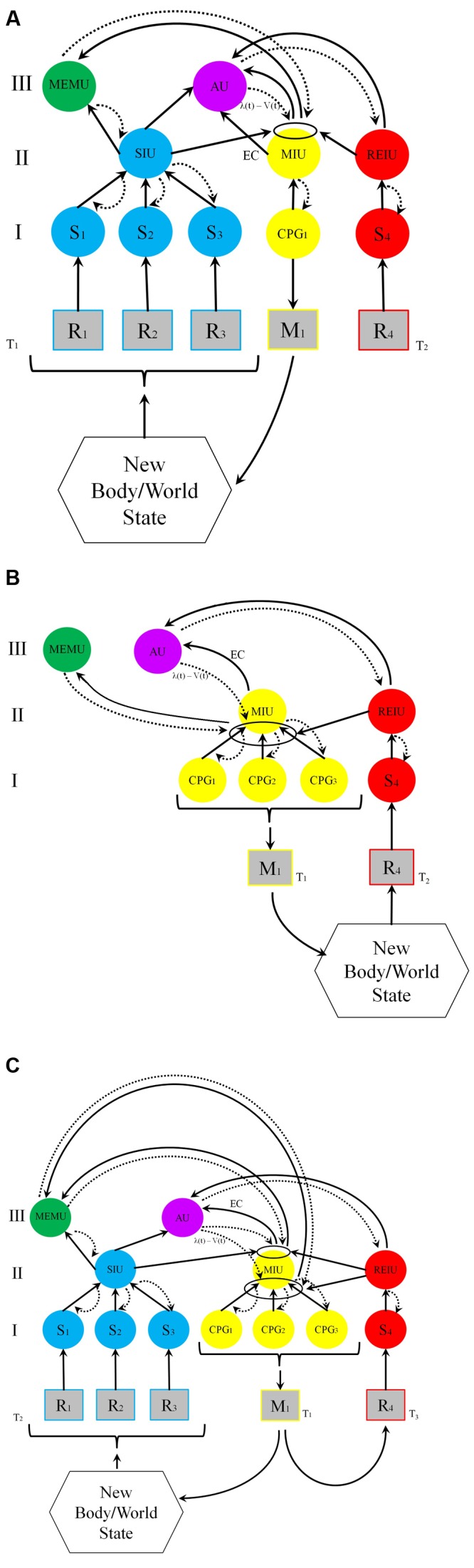
**Models of first-order UAL.** Functional units are depicted as circles; receptors and effectors are depicted as squares. Solid arrows denote bottom-up and lateral interactions; dashed arrows denote top–down interactions. The ellipses are the local loci of conjunction. I, II, and III are distinct hierarchical functional levels. R, receptor (e.g., retinal cell); M, effector (e.g., Muscle); S, primary units processing sensory information; CPG, Central Pattern Generator; SIU, Sensory Integrating Unit (all sensory exteroceptive and interoceptive information); MIU, Motor (action) Integrating Unit proprioceptive and interocpetive information); REIU, Reinforcing Integrating Unit; AU, Association Unit (motor-sensory integration); MEMU, dedicated Memory Unit. **(A)** World learning. Following an animal’s activity (e.g., saccades and exploratory touch) at time T_1_, a compound CS (e.g., a black, round and rigid ball) is processed in the sensory integrating unit, SIU. The CS comprises several perceptual elements received by receptors R_1,2,3_ and processed by sensory neural units S_1,2,3_. The construction of the compound stimulus (in the SIU unit, blue) is obtained via hierarchical predictive coding: the novel aspects of bottom-up information (depicted by ascending solid black arrows) are combined with top–down predictions (depicted by dashed curved arrows), based on available perceptual information in SIU, and on mnemonic and contextual information in the memory unit, MEMU (green). At T_2_, a reinforcing stimulus (a US such as food) activates the reinforcing unit, REIU (red), via receptor R_4_ and sensory unit S_4_. The SIU, MIU and the REIU systems activate the association unit, AU (violet), that constructs the updated model of the world at T_2_, and sends a PE to the local association unit (the ellipse). The PE is based on the discrepancy signal sent from the SIU and the EC (efference copy) signal sent from MIU [PE is the difference between the received reward λ, and the reward prediction V at time T_1_, λ(t) – V(t)]. When the discrepancy is non-zero, the strength of the association between the SIU and MIU will increase in proportion to the magnitude of the prediction error. This association circuit reconstructs engrams in MEMU. When the REIU activates the motor integrating unit MIU, it sends a signal to the effector, leading to the response via the activation of a lower-level central pattern generator unit (CPG_1_), which activates the effector M_1_. On later encounters with the stimulus, the SIU-MIU relation is primed for activity by the MEMU engrams. As a result, the compound CS will now elicit the adaptive M_1_ activity. **(B)** Self-learning. At time T_1_, a compound action carried out by M_1_ (e.g., pressing a button, turning a dial, and jumping) is originated by unit MIU, based on temporal and spatial combinations of various action patterns (implemented by central pattern generators, CPGs). This compound action pattern, which is influenced by past behaviors (engrams in MEMU), has reinforcing effects depending on present context, and is memorized both at the level of the local circuit (ellipse) and in the dedicated memory unit MEMU. As in A, the relation between the compound motor pattern and the reinforcer is processed in AU, which send a PE to the association locus. **(C)** Combination of self and world learning, typical of most classical and operant conditioning. The action results in an altered world-state, which in turn leads to the perception of a compound CS at time T_2_ (same process as in A). If at time T_3_ reinforcement of both compound movement pattern and compound percept occurs, prediction-errors that are sent to the association loci between MIU and SIU are strengthened, and both the compound CS percept the compound action pattern directed toward it will be learned.

The models depicted in **Figure 1** attempt to unify these considerations and to schematize the functional architecture of an animal exhibiting UAL by showing the relations between these three types of functions in the context of associative learning. Both models – **Figure 1A** depicting idealized world-learning and **Figure 1B** depicting idealized self-learning – have the same architecture, the difference between them being that in **Figure 1B** learning starts with reinforced motor exploration, while **Figure 1A** assumes that although action initiates perception the action is not reinforced. **Figure 1C** depicts the typical situation in which both self and world learning occur. Note that the units in the model are described at the functional level, and are not necessarily distinct anatomical units. It is possible, at least in some cases, that several functions could rely on partially, or even entirely overlapping neural structures, so that one neural unit can implement more than one functional system. For example, the recognition and memorization of compound smells processed in the mushroom bodies of insects is implemented through associations between valence (dopaminergic) signals, sensory units and integration units (Stopfer, 2014).

The basic functional architecture presented in **Figure 1** is, of course, only a schematic and simplified representation of a system that can implement UAL, and there are many properties that it *does not* show. First, it does not show second-order conditioning, although the model can readily account for such conditioning. Since many stimuli can lead to the excitation of SIU, a second compound CS can become primed for activity by the original, already reinforced compound CS, providing that the first and second compound stimuli are temporally contiguous and a non-zero PE is generated. Second, an actual biological system – an animal’s brain – is made up of more levels of integration for processing compound stimuli. (What is a compound at one level is a component of a compound for a higher level.) Mapping – of the external world, the body, and action – has more levels than suggested by our simple illustration. Third, the capacity for trace conditioning, whereby the US follows the CS after a minute or more is not explicitly depicted in the model. Fourth, many “background” factors such as the constraints imposed by the morphology of the body, the role of epigenetic memory within neurons and the neuro-hormonal and neuro-immunological interactions that shape reinforcement and integration, are not explicitly included in the model (Bronfman et al., 2014).

Each of the high-order functional systems (II and III in the figure) integrates and computes vast amounts of information. The SIU system can be considered as a model of the extra-brain environment (world and body) and the MIU system as a model of prospective actions (that interacts with a model of the body). These systems are influenced by the outputs from the MEMU that reflect the animal’s past history, and outputs from the current homeostatic state monitored by the REIU. The REIU assigns reinforcement value to percepts and actions according to the deviation of the system from a state of homeostasis computed by the AU system. So for thirsty, dehydrated animal water is a strong reinforcer whereas for one that is fully hydrated it is not. Similarly, complex actions require a model of prospective movements that provide inputs to a model of the animals’ body. The proprioceptive and perceptual body models interact (e.g., through reafference) as the animal responds to the model of the external world that it had constructed. All these are modulated, and, in turn, modulate, the compound memory unit. This is necessary if a mobile animal is to react to the world in a manner that goes beyond learning through sensitization, habituation and limited associative learning. Note that in addition to the effects of binding in the SIU and MIU systems that affect the construction of engrams in MEMU, the association system, which represents the relationship between SIU and MIU, reconstructs corresponding engrams in MEMU as well. UAL therefore entails the coupling of several functional systems that are influenced by the animal’s past history and that are represented in (one or more) dedicated memory units.

The UAL model can be readily reformulated within the general predictive coding framework suggested by Friston (2009). A prior is a top–down signal, shaped by the animal’s evolution and past learning (e.g., descending signals from SIU to S’s), which constrains and modifies the ascending signals of a lower unit. The ascending signals – when different from the learned state – can be interpreted as “PEs” (e.g., ascending signals for S’s to SIU). The reliability of the PEs (termed “precision”) is calculated at each level through lateral inhibitions of alternative stimuli-response relations (these are not shown in the model), which prevent the blurring of the PEs by other competing signaling circuits.

The neural-bodily architecture that this requires has been only partially deciphered in different animal taxa. Nevertheless, some neural structures that implement this functional organization have been identified in vertebrates, many arthropods and some mollusks (**Table 1**). In mammals and other vertebrates, several structures of the cerebral cortex and of the midbrain (including the colliculi, the hippocampus, basal ganglia and hypothalamus) and several additional subcortical areas, including the amygdala and the thalamus, are crucial for compound perception and action as well as for assigning overall value and motivating the animal to act (Merker, 2007). Similarly interacting brain centers, analogous or homologous to these vertebrate regions, are present in invertebrate phyla such as arthropods and some annelids (for details see Tomer et al., 2010; Strausfeld and Hirth, 2013; Barron and Klein, 2016; Klein and Barron, 2016).

**Table 1:** Brain structures implementing the functions of minimal consciousness in vertebrates, arthropods and mollusks.

		Integrating into compound patterns (correspond to SIU, MIU, AU)		Globally acting value mechanisms and factors (correspond to REIU)	Memory for compound patterns (correspond to MEMU)
				
		Exteroceptive (perception of external world and of body parts)	Proprioceptive (movement of body in space)	
Vertebrates		Cortex, superior and inferior colliculi, cerebellum	Superior colliculus, cerebellum	Cortex, basal ganglia (nucleus accumbens andventral striatum), cingulate cortex, amygdala, reticular formation, subtantia nigra, thalamus, periaqueductal gray, hypothalamus, mammillary bodies, pituitary; dopamine	Cortex, hippocampus, basal ganglia, cingulate cortex, fornix, mammillary bodies, cerebellum

Arthropods	Insects	Mushroom body, central complex	Central complex	Lateral accessory lobe, and perhaps also the MB and the central complex (FB and EB), in which there are dopamine receptors; specific neurons; dopamine and octopamine	Mushroom body, central complex

	Crustaceans	Hemiellipsoid body	Central complex	Central complex; octopamine and serotonin	Hemiellipsoid body, central complex

Mollusks	Cephalopods (e.g., octopus)	Superior frontal lobe, vertical lobe, and peduncle	Brain and peripheral nervous system	Vertical lobe; octopamine and serotonin	Superior frontal lobe, vertical lobe


*The information in this table relies, primarily, on Strausfeld and Hirth (2013), Barron and Klein (2016), Feinberg and Mallatt (2016), and Wolff and Strausfeld (2016). For more detailed information on arthropod central complex, see Loesel et al. (2002), Homberg (2008), and Pfeiffer and Homberg (2014). The information on value mechanisms and factors in invertebrates relates to reinforcement and reward learning in these animals. It is based, in general, on Barron et al. (2010), and on additional sources for individual species. For a specific insect neuron (VUMmx1) that serves the function of a value system, see Menzel and Giurfa (2001), and for specific dopamine neurons in *Drosophila*, see Hige et al. (2015), described in detail in Poo et al. (2016, p. 16), for biogenic amines and behavioral reinforcement in crustaceans, see Kaczer and Maldonado (2009), Kravitz and Huber (2003). Finally, for biogenic amines involved in reinforcement learning in the octopus, see Shomrat et al. (2015). Benny Hochner (private communication, 20th August 2016) provided the information regarding the value system of the octopus. Note that some functions can be implemented by the same anatomical structures or circuits.*

The largest part of the insect brain, the protocerebrum, contains the mushroom bodies (MB) and the central complex (CX), which is believed to be homologous to the hippocampus and basal ganglia in vertebrates and are necessary for the storage of compound patterns and for integrated perception and motivation. Similarly, the hemiellipsoid bodies of crustaceans are functionally homologous to the hippocampus (Wolff and Strausfeld, 2016). In cephalopod mollusks such as octopus and cuttlefish, two brain structures, the vertical lobe and the superior frontal lobe, form complex networks that are jointly analogous to the vertebrate hippocampus. These integrating brain structures serve as the basis for cephalopod learning and memory (Shomrat et al., 2011; Hochner, 2013). In gastropod mollusks the interactions between sensory integrating units, memory units for the storage of perception and action-patterns, and a flexible value system, do not seem to form similar multilevel processing. However, we believe that the terrestrial gastropods *Helix* and *Limax* might exhibit UAL, since the procerebral (PC) lobes of these land snails are involved in relatively advanced forms of learning (enabling blocking and second-order AL) and the land snail olfactory memory resembles mammalian mechanisms at several processing levels (Watanabe et al., 2008). Hence, one cannot rule out the possibility that their neural architecture can support sentience. More information is needed, however, about the learning capacities of animals in these groups as well as their ability to integrate sensory stimuli and generate compound actions. In animals that can learn only on the basis of pre-existing reflexes such as the sea-slugs *Aplysia californica* and *Pleurobranchaea californica*, complex neural hierarchy has not been found and the integrative structures seem unspecialized (Gillette and Brown, 2015; Hawkins and Byrne, 2015). Hence it seems that all vertebrates, many arthropods, and some mollusks have all the neural structures necessary to support UAL. For some animal phyla we lack sufficient information about the relevant structures or have information for only some of these structures (e.g., Wolff and Strausfeld, 2016). In other phyla, such as flat worms and nematodes, there is no evidence for the presence of such structures. This is consistent with the behavioral data showing no evidence for UAL in these groups.

The existing information about the animals in taxa that show the defining attributes of UAL, or exhibit complex learned behaviors that can be considered as indicators for UAL (such as an ability to learn rules, or navigate a complex novel terrain) is patchy (see **Table 2**). The data are nevertheless compatible with the conclusions drawn from the anatomical data. Limited AL but no UAL was found in nematodes (Ardiel and Rankin, 2010). Bhatla (2014) found conditioning only when the CS and US co-occurred, so conditioning in this groups may be conditional sensitization. No UAL has been found in aplysiid mollusks (Marinesco et al., 2003; Hoover et al., 2006), flat worms (Jacobson et al., 1967; Nicolas et al., 2008; Prados et al., 2013) or annelids (Crisp and Burrell, 2009; Shain, 2009). In echinoderms elemental (limited) conditioning has been reported but the necessary controls for distinguishing AL from sensitization were not performed (McClintock and Lawrence, 1982). In cnidarians, the single experiment reporting limited conditioning (Haralson et al., 1975) was not replicated. Torley (2007) conducted a literature search supplemented by personal inquiries from leading scientists working on learning in cnidarians and was unable to find any study that demonstrated conditioning in cnidarians.

**Table 2:** Groups exhibiting UAL, or learned behaviors that can be seen as proxies of UAL.

Learning type	Phylum	References (reviews and sample original papers)^∗^
Pavlovian conditioning (involving perceptual fusion); Operant conditioning (involving novel action patterns and spatial learning)	Mollusks	BOOK: Menzel and Benjamin, 2013 (chapters 14–22 gastropods; chapters 23–25 cephalopods); BOOK: Mather et al., 2010 (octopus); REVIEW: Hochner et al., 2006 (octopus); Boal et al., 2000 (octopus); Jozet-Alves et al., 2013 (Cuttlefish) Watanabe et al., 2008 (gastropods); General BOOK: Corning et al., 1973 (vol. 2, chapter 10, vol. 3, chapter 11); REVIEW: Perry et al., 2013;
Pavlovian conditioning with compound CSs (involving non-elemental learning); Operant conditioning (involving novel action patterns and spatial learning); Conceptual learning;^a^ Number-based learning;^b^ Navigation learning;^c^	Arthropods	BOOK: Galizia et al., 2012 (part VI, honeybee); Bhagavan and Smith, 1997 (honeybee); Brembs and Heisenberg, 2001 (*Drosophila*); Young et al., 2011 (*Drosophil*a); Mizunami et al., 2013 (cockroaches); Collett et al., 2013 (mainly hymneopterans); Collett and Collett, 2009 (insects); Boisvert and Sherry, 2006 (bumble bee); Magee and Elwood, 2013 (shore crabs); General BOOK: Menzel and Benjamin, 2013 (chapter 26 crustacea; chapters 27–42 insects); BOOK: Giurfa et al., 2011 (pp. 5–101, insects); BOOK: Corning et al., 1973 vol. 2, chapters 5–9); REVIEW: Perry et al., 2013;
Pavlovian conditioning with compound CSs (including non-elemental learning); Operant conditioning (involving novel action patterns and spatial learning); Conceptual learning;^a^ Number-based learning;^b^ Navigation learning;^c^	Vertebrates	Agrillo et al., 2010 (fish); Schumacher et al., 2016 (fish); Newport et al., 2014 (fish); General BOOKS: Macphail, 1982, 1987 (all vertebrates); BOOK: Razran, 1971 (mainly mammals); REVIEW: Moore, 2004 (mainly birds and mammals); REVIEW: Perry et al., 2013.


*The literature on complex learning abilities in mollusks, arthropods and vertebrates is vast, but the number of species investigated for compound learning is a very small sample of the species in each of these phyla. We present here books and comprehensive reviews that cover much of the literature on the subject, as well as a sample of original papers. It should be noted that in some cases there is still a controversy about particular cognitive abilities of specific species. For example, there it is as-yet unsettled whether or not honeybees exhibit blocking (see Blaser et al., 2004, 2006; Guez and Miller, 2008). ^a^Includes: the transfer of a learned rule to other samples (e.g., matching a new stimulus to a previously encountered sample); comparing stimuli in different sensory modalities, and learning relations such as “same” and “different.” ^b^Ability to distinguish between the number/amount of cues presented. ^c^A combination of classical conditioning of landmark cues and operant conditioning of proprioceptive cues (e.g., step “counting”).

## The Attributes of UAL Instantiate the Properties of Minimal Consciousness

Based on the behavioral and functional characterization of UAL presented in the previous section, we suggest that the properties listed in section 1 are also individually necessary and jointly sufficient for the construction of a UAL system.

(1) *Flexible value systems and goals*. Although any form of associative learning entails values and goals, with UAL these become extremely flexible and variable. Through perceptual fusion all stimuli can be associated with reinforcement, and through cumulative (second-order) learning, the organism’s value system is recursively adjustable: following experience, numerous stimuli can serve as potential USs, signaling value in subsequent learning. The flexible value-reinforcement system must provide a common currency of *overall* sensory evaluations for reward and punishments to enable motivated behavior. As a result, the animal’s behavior becomes directed to many more goals than that of an animal lacking UAL. In addition to modifying its behavior in light of phylogenetically inherited goals (arrived at through classical reflexes and innate exploratory behaviors), the animal can acquire new goals and sub-goals during ontogeny.
(2) *Unity and diversity through feature integration (binding)*. By definition, UAL requires binding. Since with UAL specific configuration of features can be learned, this suggests that UAL must rely on neural mechanisms that enable integration, identification and discrimination among compound stimuli that differ in their specific conjunction of underlying features within and across modalities (Razran, 1965; Over and Mackintosh, 1969; Bellingham and Gillette, 1981; Edwards et al., 1982; Colombo and Graziano, 1994; Watanabe et al., 1995). As suggested long ago, discrimination is tightly related both to consciousness (e.g., James, 1890; Edelman, 2001; Crick and Koch, 2003) and to the evolution of complex forms of learning (Wells, 1968; Razran, 1971). Selection for increased discrimination ability probably drove the evolution of binding. In addition to perceptual binding, UAL entails binding of action-patterns, which are learned, as a whole, rather than each action separately.
(3) *Global accessibility of information*. With UAL, a compound stimulus is associated with reinforcement via hierarchical bi-directional interactions, such as those implemented by predictive coding. These dynamics entail complex, reciprocal flow of information, in which bottom–up sensory information is compared with top–down “expectations” (see **Figure 1**). The top–down “expectations” are by definition global (non-local), as they depend upon more than a single input, and similarly, affect more than a single specific output and exclude the effects of others.
(4) Unity through integration and exclusion, and global accessibility are the features of consciousness highlighted by Tononi’s information-integration theory (IIT; Tononi, 2004; Tononi et al., 2016). According to the IIT, the degree to which information is integrated in the system (across the system’s sub-components) above and beyond the sum of information integrated at each sub-component, reflects the system’s consciousness level (measured by an index termed Φ). Compound pattern formation and hierarchical predictive coding are prominent examples of information-integration dynamics. However, whereas a high level of information-integration may be a necessary feature of consciousness, it is not a sufficient condition for it. A *specific type* of integration is necessary for sentience (we suggest that it is the type of integration presented in **Figure 1**). Additional properties such as goal-directedness and flexible values that take into account the physiological and external context (and further properties listed below) must be *explicitly* considered as well.
(5) *Temporal thickness*. The formation of compound percepts via hierarchical predictive coding entailed by UAL, requires temporal durability because the multi-directional reverberating processes described in **Figure 1** are relatively time-consuming as compared to “flat” feed-forward processes. This renders percept-formation during UAL temporally “thick.” Visual binding occurs at a (relatively) late processing stage (Bodelón et al., 2007), and the increased temporal durability of compound percepts based on back and forth (reentrant) signaling is the basis of working memory (Wang, 2001) and of trace conditioning (in which there is a temporal gap between the US and the CS; Solomon et al., 1986; Woodruff-Pak and Disterhoft, 2008; Dylla et al., 2013).
(6) *Selection and attention*. Whereas any form of learning is, by definition, selective (Changeux et al., 1973; Changeux and Danchin, 1976; Skinner, 1984; Kandel, 2007), UAL, requires a unique, complex mode of selection in which compound information is learned, and learning occurs only when the compound information signals unexpected reward (as in prediction-error learning). This constraint implies that most of the time learning is inhibited, since many of the stimuli which the animal perceives, though perfectly associated with the US, are not “surprising” (as with blocking; see Kamin, 1969). A related requirement if for top–down attention, which is considered by many to be necessary for integrating multi-feature stimuli into a compound (Treisman and Gelade, 1980; Huang and Pashler, 2007).
(7) *Intentionality (Aboutness)*. Predictive-coding is intentional – it is “about” the world and the body since it requires models of the world, of the body, and of action, and is based on mapping/modeling-relations between sensory signals and their latent external causes (Summerfield et al., 2006; Friston, 2009; Clark, 2013; Hohwy, 2013). UAL, which necessitates and encompasses such modeling, ties the models with the organism’s goals, as reflected by its bodily states and motor activities, and is therefore intentional par excellence (Brentano, 1874; Searle, 1983; Edelman, 2003; Freeman, 2003).
(8) *Self and embodiment.* UAL instantiates the philosophical notion of self. “Minimal self,” as most clearly described by Metzinger (2009) and Merker (2005, 2007, 2013), requires a model of the integrated yet rapidly changing world in which a model of the coordinated and flexibly changing and moving body is nested. Such a nested model provides a stable updateable perspective that enables the flexible evaluation of the various changes in the world–body relations. This notion of self can only be achieved via hierarchical, multilayered predictive-coding, allowing the animal to distinguish between the effects of compound self-generated and world-generated stimuli. Furthermore, comparing the actual sensory consequences of the animal’s complex motor commands to those of the sensory feedback that is predicted by its self-model allows the animal to “predict” the effects of its own actions (Holst and Mittelstaedt, 1971; Brembs and Heisenberg, 2000; Jeannerod and Arbib, 2003; Brembs, 2008). The formation and maintenance of complex body and world models, and the distinction between self-produced and world-produced sensory inputs, involve the integration of external-world-derived and internal-world-derived stimuli along with action patterns that are constantly updated, evaluated, and compared with past learned memories (Botvinick and Cohen, 1998; Ramachandran, 1998; Tsakiris and Haggard, 2005; Blanke, 2012; Seth, 2013). The interactions between the integrating units presented in **Figure 1** constitute such a self-model.

In summary, UAL seems to require an enabling system with the properties and capacities that are deemed jointly sufficient for minimal consciousness. Animals with an integrated body, actions, and world models, that distinguish between self and world and act according to reinforcement signals based on these integrations in multiple domains of sensation and action, can be said to have a self, and the nested neural representations they form seem necessary for generating the integrated sensory states that we call subjective experiences. Although UAL *is not* to be equated with subjective experiencing (just as a DNA replicating sub-system cannot be said to be living), a UAL architecture *in a biologically evolved system* can be the basis for the reverse engineering of an experiencing system. This is analogous to a DNA replicating subsystem which can be the basis for reverse-engineering its enabling system, a living cell that includes metabolic cycles and membrane-synthesis mechanisms. However, a genetic algorithm that employs recombination and mutations in long strings of digits, though implementing unlimited heredity, does not render the computer chips within which it is implemented alive. Like the cell, the UAL enabling system in an animal includes many crucial additional components such as immune and hormonal factors and bioelectric fields that unify the entire system at several spatial and temporal scales and that must be constituents of a reverse engineered minimally conscious system. Such factors may not be necessary in an artificial system, and we are therefore not suggesting that UAL is a sufficient condition for minimal consciousness in artificial systems (see Verschure, 2016 for an insightful discussion of the possibility of consciousness in artificial systems), though we regard it as a sufficient condition in biologically evolved ones.

Although our suggestion that UAL can serve as an evolutionary marker of minimal consciousness does not imply that we have solved the “hard problem” (Chalmers, 1997), our model can contribute to the elucidation of the dynamics of minimal consciousness. We believe that our proposal provides an evolutionary-selective rationale for the emergence of the structures and processes suggested by prominent models of consciousness: Merker’s (2005, 2007, 2013) theory that focuses on the interactions between the neural processes underlying target selection, action selection and motivation; Changeux and Dehaene’s Global Neural Workspace Model (Dehaene et al., 1998; Dehaene, 2014) that emphasizes the integrative interactions between perceptual, motor, attentional, memory and value systems; and the Dynamic Core theory of Edelman (2003) that underscores value systems, multi-level mapping and integrative processes that relate compound inputs to past learning responses and future needs.

As we show in the next section, our suggestion that selection for UAL led to the evolution of minimal consciousness and that UAL is its evolutionary transition marker may have important implications concerning the distribution of sentience in the living world and concerning research on animal consciousness. Before we briefly discuss these implications, however, we must clarify an issue that is a common source of misunderstanding and describe how our perspective compares with other frameworks that aim to account for phenomenal consciousness.

Crucially, a capacity for UAL is a *positive marker* – its presence can tell us that an animal is endowed with sentience, but from its absence we cannot deduce that an animal is non-sentient. Humans who are incapable of UAL, such as a neonates or hydroanencephalics, can nonetheless be sentient (Merker, 2007), because they are equipped with a preexisting, evolved and functional, UAL-supporting mechanisms that are in place even when they are incapable of UAL for developmental or pathological reasons. Just as a cell is considered alive even after its nucleus has been removed (and hence it is lacking an unlimited heredity system) but all or most of the other systems that had evolved in the context of the evolution of unlimited heredity are functioning, so an animal lacking a current capacity for UAL (but exercising the capacities that were selected during the evolution of UAL) can be considered sentient. Our argument is that the *evolutionary origins* of UAL and its enabling system were coupled and therefore that an organism with an evolutionary capacity for UAL and an evolved enabling system will manifest consciousness as well. Our answer to the question whether animals with some but not all the components of UAL have some level of sentience is that sentience will not be evolutionarily sustainable in such animals since positive selection for UAL is required for its maintenance. Just as a proto-cell with a limited inheritance system may (arguably) be considered alive but will go extinct if an unlimited heredity does not evolve, so a system that has lost its capacity for UAL, will fail, over evolutionary time, to sustain sentience. Moreover, our theory does not suggest that unconscious processing of information is impossible once the organism has the capacity for UAL. Rather, we predict that such unconscious processing will not have the potential to yield UAL.

While our approach builds on many current frameworks for understanding minimal consciousness, it is also distinct from them. The UAL functional architecture is, either implicitly presupposed by several leading models of consciousness or is compatible with them. For example, the global neural workspace model (GNW) suggests that the perceptual, motor, memory, value and attention systems come together to construct mental states (Dehaene, 2014). These very same systems are part of our UAL architecture, although in our model the coupling among them is explicit: we specify the temporal and functional relations between the relevant subsystems, with attention instantiated by the precision of evaluating discrepancies, through predictive coding. Similarly, our UAL model is compatible with Gerald Edelman’s dynamic core model, which focuses on value systems and multi-level mapping and integration processes that relate compound inputs to learned responses and anticipated (future) needs (Edelman, 2003). These dynamics are explicitly accommodated by our model, which, in addition, specifies the type of functional relations that can instantiate these dynamics.

A more recent model, Tononi’s integrated information theory (IIT) of consciousness (Tononi et al., 2016), posits that consciousness is based on composite, integrated (irreducible to non-interdependent subsets), intrinsic cause-effect processes, which exclude a lot of alternatives. This integrated information (Φ) can be measured. Tononi focuses on the unity and global accessibility aspects of consciousness (other features of consciousness are seen as derivable from or implied by his theory). Our suggested UAL architecture requires composition, integration and exclusion of information as well, but our model also explicitly includes other aspects of minimal consciousness, such as the role of reinforcement and memory for compound patterns.

Our UAL model is also strongly related to models that emphasize the notion of an action-constructed bodily self. The emotion-focused theory of Damasio highlights the perceptions that the organism has of its own body through the continuous monitoring of its internal state by the brain, which is represented when objects in the external world modify this internal perception (Damasio, 2010). In our model, the interactions of the REIU system with the SIU and MIU systems (which are affected by the MEMU) are compatible with Damasio’s suggestions. In the UAL model, hierarchical representations are built into the *specific* patterns of interaction between lower and higher sensory and motor units.

Merker’s (2007, 2013) model of consciousness, is perhaps the closest to our UAL model. His tripartite target selection, action selection and motivation are operationalized by our integrating units and our REI unit. The motion-stabilized body-world interface organized around an ego-center that Merker suggests is, in our model, the outcome of a temporally specific efferent-copy signaling. The stable perspective that the animal adopts also depends on efferent copy signaling, as well as on continuous updating of expectations via predictive coding.

Although compatible with Feinberg and Mallatt’s focus on hierarchically organized neural maps, our account of minimal consciousness and mental states is *incompatible* with their distinction between separate types of affective, exteroceptive and interoceptive consciousness (Feinberg and Mallatt, 2016; for a similar distinction see Panksepp, 2005). According to our view, *all* conscious states are sensory, *all* involve interactions with the motor system, *all* involve memory for compound patterns, and *all* are valued/stabilized. There are, of course, conscious mental states that are primarily visual, auditory, or tactile, or experiences that stem from responses to stimulations of receptors within the body and changed states of the CNS, resulting in internal pains, anxiety, imbalance and fatigue, but these distinctions do not entail separate types of consciousness. All these experiences share the same basic pattern of interactions depicted in the UAL model. Clearly, the source of the sensory stimuli may be different under different ecological conditions and for different animal taxa, and the relative importance of the different integrating units that are involved in processing particular experiences is therefore likely to be different too. Although there are clearly many different mental states, we believe that the idea that there are several types of consciousness is an error stemming from the attribution of consciousness to parts of the systems (to the activity of the REIU, MNU and SIU) rather than to the activity of the system as a whole.

## Implications and Future Directions

A characterization of UAL at the functional–behavioral level along the lines suggested in this paper, leads to a variety of testable questions about the relation between consciousness and learning. Our model predicts that first and second order learning about novel compound percepts and actions requires sentience. This is supported by the finding that masked, unconsciously perceived and novel CSs, such as pictures of flowers or mushrooms do not give rise to conditioning, while masked angry faces do (Öhman and Soares, 1993). We hypothesize that the level of perceptual integration of novel compound percepts is too low to form an engram in the MEMU system, while preexisting engrams of angry faces (or, we predict, an *already* consciously learned compound) can be primed by even degraded signals. Studies of this type need to be extended to test for second order conditioning of novel and already-learned compound stimuli and actions in both humans and animals (whose ability to become conditioned to masked stimuli of different types was not investigated). Second, we can test when during human ontogeny UAL emerges, how language influences it, and if there are lesions or other pathologies that interfere with it (some studies begin to answer these question; see for example Kawai et al. (2004), for tests of limited associative learning already in chimpanzee fetuses, and evidence, for example by Winocur and Eskes (1998) and Wang et al. (2015), that lesions of the prefrontal cortex interfere with complex modes of associative learning).

One important potential implication of our theory is that it provides a theory-driven, explicit prediction regarding the distribution of minimal consciousness in the animal kingdom. The status of this question in the consciousness literature is at present wide-open (see Liljenström and Århem, 2008 for an exposition of the different views). Suggestions range from attributing consciousness to linguistic animals (i.e., humans) alone (Macphail, 1987) to all living creatures (Margulis, 2001) and all matter (Chalmers, 1997).

If we accept that UAL is an evolutionary marker of minimal consciousness, we can investigate its existence in the animal kingdom and by extension address the distribution question. A survey of the distribution of associative learning (both limited and UAL) in the animal world suggests that this mode of learning is found in at least six, possibly nine, different phyla (Corning et al., 1973; Perry et al., 2013) and that limited AL probably first evolved in the early Cambrian 540 million years ago (Ginsburg and Jablonka, 2010a,b). The distribution of UAL has been less systematically studied, but what we do know (see **Table 2**) suggests that it evolved in the arthropods and the vertebrates during the middle Cambrian period, shortly after these groups evolved, and much later (about 250 million years later), in the mollusks. If we are correct and UAL conferred a significant evolutionary advantage during the Cambrian, we expect that species diversification in the fossil record would be particularly evident in phyla of animals exhibiting UAL and interacting with UAL animals.

We have suggested that increasingly open-ended associative learning and minimal consciousness not only evolved during the Cambrian, but that AL evolution itself may have been one of the factors that drove the Cambrian explosion (Ginsburg and Jablonka, 2007a,b, 2010a,b, 2015). The first part of our suggestion, the idea that minimal consciousness emerged during the Cambrian, is gaining support: According to Merker’s model of the self, all vertebrates have minimal consciousness (Merker, 2007, 2013). Since this phylum evolved in the Cambrian, minimal consciousness must have evolved in that taxon during that era. This line of reasoning has now been extended to arthropods by Barron and Klein (2016; Klein and Barron, 2016), who explicitly suggest that the minimally conscious self originated in the Cambrian in both vertebrates and insects. The same conclusion has been reached by Feinberg and Mallatt (2013, 2016) on the basis of their work on the brain structures and organization supporting the hierarchical mapping of the world and the body in vertebrates and in some invertebrates, as well as by Godfrey-Smith (2016) who focuses on cephalopod consciousness. There is, thus, is a small but growing convergence on a view that minimal consciousness first evolved, in parallel, during the Cambrian era in two animal phyla. Our second proposal, that the evolution of learning was one of the factors that drove the Cambrian explosion is at present still under-researched.

A related issue is linked to debates about the kind of primary sensory experiences: were visual experiences primary? Or maybe olfactory experiences came first? Or were the first experiences interoceptive-affective? From our perspective, these debates miss the integrative nature of all experiencing. Since movement and valued sensory inputs are always involved in the construction of consciousness, sensory signals from the surface of the moving body, proprioceptive signals, and interoceptors’ signaling, have contributed to *all* mental states. The relative significance and richness of the exteroceptive signals in particular lineages depended on the evolution of sensory receptors and processors of that lineage, which was intimately related to its specific habitat. A sense of balance and the ability to detect vibrations were probably important in all moving animals, while in animals living close to the water surface, eyes and visual experiences were probably of special significance, and olfactory experiences may have played a larger role in animals that moved on the bottom of the sea where little light penetrated. Whatever the sense/s most abundantly employed, it seems likely that all existing types of exteroception and interoception contributed to the ability of UAL animals to construct a multimodal representation of its “self” in the world. In all these animals, the default, spontaneous exploratory activity that led to distinction between self and world was rewarding – maybe even joyful, as Humphrey (2011) suggested – encouraging the animal to engage with its world.

The view we put forward has implications for the ongoing debate about the function of consciousness. The usual argument about the teleo-functions of consciousness is that since being conscious originated at some point in the history of life and has been maintained in at least some lineages, it stands to reason that it has some functions. Seth (2009) reviewed various ideas about the functions of consciousness and pointed to a common thread in the thoughts of both philosophers of mind and cognitive scientists: the function of consciousness is to integrate information, resolve ambiguities, and enable novel, flexible and goal-directed behavior; following Morsella (2005) he called this broad, shared agreement, the “integration consensus.” Since our argument was that a system enabling UAL entails the integration of information, leads to a massive increase of discrimination, and allows the generation of flexible goal-directed behavior, does it follow that UAL is the “true” function of minimal consciousness?

The perspective we present in this paper suggests that asking what is the function/s of consciousness is a misleading question for the very same reason that asking what is the function of life is misleading. Life has a goal, but no function: with the emergence of life, a new teleological (goal-directed) order emerged. While the subsystems of a living system like a cell have distinct activities (e.g., metabolic cycles transform energy), these processes can be considered functions only when they conjointly subserve the goal of “survival and reproduction,” that is, when they dynamically organize and enable the survival and reproduction of the cell as a whole. The notion of biological teleo-function, ascribed to parts or processes that comprise an entity, is, by definition, only understandable within the contexts of a goal-directed system. We suggest that consciousness, like life, is best regarded as a goal-directed dynamic system rather than a functional trait. Just as life, in which functions such as membrane-maintenance and metabolism serve a telos of survival and reproduction, the functions that operate within the framework set up by consciousness, such as UAL (which involves perception, action selection and motivation) serve a telos of ascribing values to ontogenetically encountered and constructed compound objects, actions or states.

Our approach also has ethical implications. Whereas some biologists infer that animals like fish do not suffer pain because they do not have a mammalian-like neo-cortical representation of nociceptive stimuli (Rose, 2002; Key, 2016), like Merker (2016) and many other biologists, we argue that sentience does not require a mammalian neo-cortical organization. Clearly, however, if an animal either lacks particular sensory receptors, organs, or a neural architecture that leads to the processing of specific inputs as conscious experiences, we do not expect the animal to be conscious of these sensory inputs. However, given our profound ignorance about most animals’ nociception, we should prefer to err on the side of caution.

## Conclusion

We offer a novel evolutionary perspective for studying consciousness that proved successful when applied to the study of life. Based on this heuristics, we suggest that UAL is the evolutionary transition marker distinguishing between organisms lacking consciousness, and minimally conscious, or sentient, organisms. We describe the properties and functional organization of UAL, the neural substrates underlying it and its distribution across the animal kingdom. We show that the processes, structures and dynamic organization that enable UAL, constitute an integrated functional system whose attributes satisfy the list of properties that have been recognized as individually necessary and jointly sufficient for instantiating minimal consciousness in animals. We suggest that the evolution of UAL was the selective context in which minimal consciousness emerged, provide testable empirical predictions that are derived from our model, and propose that consciousness first emerged during the Cambrian era in arthropods and vertebrates and about 250 million years later in cephalopods. We see UAL as one of the functions that minimal consciousness, an ontogenetic goal-directed system, had afforded, and suggest that the emergence of consciousness led to the generation of a new order of things, to a new functional realm, altering the very notion of being.

## Author Contributions

All authors listed, have made substantial, direct and intellectual contribution to the work, and approved it for publication.

## Conflict of Interest Statement

The authors declare that the research was conducted in the absence of any commercial or financial relationships that could be construed as a potential conflict of interest.
